# Heterogeneous Sensing Data Analysis for Commercial Waste Collection

**DOI:** 10.3390/s20040978

**Published:** 2020-02-12

**Authors:** Foued Melakessou, Paul Kugener, Neamah Alnaffakh, Sébastien Faye, Djamel Khadraoui

**Affiliations:** Luxembourg Institute of Science and Technology (LIST), L-4362 Esch-sur-Alzette, Luxembourg; paul.kugener@list.lu (P.K.); neamah.alnaffakh@list.lu (N.A.); djamel.khadraoui@list.lu (D.K.)

**Keywords:** smart waste collection, waste monitoring, wireless sensor networks, LPWAN, data analytics, machine learning, cluster analysis

## Abstract

Waste collection has become a major issue all over the world, especially when it is offered as a service for businesses; unlike public waste collection where the parameters are relatively homogeneous. This industry can greatly benefit from new sensing technologies and advances in artificial intelligence that have been achieved over the last few years. However, in most situations waste management systems are based on obsolete technologies, with a low level of interoperability and thus offering static processes. The most advanced solutions are generally limited to statistical, non-predictive approaches and have a limited view of reality, making them weakly effective in dealing with day-to-day business issues (overflowing containers, poor quality of service, etc.). This paper presents a case study currently being developed in Luxembourg with a company offering a business waste collection service, which has a significant amount of constraints to consider. Our main objective is to investigate the use of multiple waste data sources to derive useful indicators for improving collection processes. We start with company-owned historical data and then investigate GPS information from tracking devices positioned on collection trucks. Furthermore, we analyze data collected from ultrasonic sensors deployed on almost 50 different containers to measure fill levels. We describe the deployment steps and show that this approach, combined with anomaly detection and prediction techniques, has the potential to change the way this business operates. We also discuss the interest of the different datasets presented and multi-objective optimization issues. To the best of our knowledge, this article is the first major work dedicated to the world of professional waste collection.

## 1. Introduction

A smart city is a municipality that uses smart infrastructure (i.e., information and communication technology) to improve the effectiveness of government and business services, and citizen welfare. These services include, for example, intelligent transportation systems, smartphone detection, traffic congestion, smart roads, and finally such as in our use case, waste management. Conventional waste collections are complicated and costly (i.e., highly resource-intensive). A fleet of trucks drive along busy streets on an arbitrary schedule using inefficient routes and may visit dumpsters that are completely empty, or on the contrary over-filled. Conventional waste collections are based on a lot of speculation where the filling level of bins could vary, ranging from overflowing, partially filled, to completely empty. This would result in unnecessary consumption of city resources and fuel. The most recent study by Annenberg Learner Foundation [1] highlighted the annual growth of producing rubbish in the USA, where each individual generated around 4.6 pounds each day (which is resulted in a total of 230 million tons per year). Such an increase in environmental issues (including problems with waste collection, transport, processing, and disposal) have been considered to be a serious situation.

The rapid development of sensing technologies opened great opportunities for researchers and waste management companies to design and develop various platforms to optimize the time and resources allocated to waste management. Smart Waste Collection (SWC) is now attracting considerable attention given its integral part of any smart city. It is expected that the revenue of SWC systems will reach 600 million in 2020 (i.e., 9.2% of Compound Annual Growth Rate [2]). Such systems have several advantages over the conventional waste collection—for example, minimizing the pickup point would result in dispatching fewer vehicles (i.e., route optimization), reduction in cost, traffic, congestion and CO2 emissions, and enhancing customer satisfaction.

Existing smart waste management platforms such as Sensoneo [3], CleanCUBE [4], and Bigbelly [5] were introduced to improve the operational performance and reduce the cost expenses that are resulted from the traditional trash collection processes. Nevertheless, the proposed solutions are limited to the management of solid waste for public markets (e.g., for the city council and householder), and using specialized and costly equipment (i.e., trash compactor). Therefore, it is essential to automatically collect and process ambient waste data (e.g., fill level and weight of the containers, and truck route coordinates) rather than using manual, limited, and inaccurate information collected by the driver. To this end, this paper presents an intelligent platform called SWAM [6] (Smart WAste Collection SysteMs) that aims to offer online dynamic scheduling and routing of the collection trucks (i.e., optimizing the route schedule, and frequency of trash collection for businesses). More information about the platform can be found via [7], which presents a view of the different modules, while a small-scale demonstration of the project is presented in [8].

The proposed platform was deployed in Luxembourg—a small and very dynamic country, where the impact of a solution can be easily measured and subsequently reproduced on a larger scale. More specifically, the work presented in this paper focuses on the SWAM Data Management System, which aims to collect and process multiple data flows in collaboration with a local waste company in Luxembourg, namely Polygone (PoL) and external services, including:

- Business data flows composed on the one hand of data imported from PoL’s Enterprise Resource Planning (ERP, e.g., customer inventory) and on the other hand of needs and constraints reported by PoL’s customers and defined according to their socio-professional categories.
- Waste bin filling levels, regularly transmitted by ultrasonic sensors to be deployed as part of the SWAM project.

This paper presents a thorough analysis in the following manner:

- Collecting GPS information (trucks fleet) for real time monitoring.
- Deploying ultrasonic filling detection sensors in several bins to capture real life data.
- Proving a comprehensive analysis of the current state of the art in the area of SWC and a technology evaluation to determine the basis for such an approach.
- Proposing and validating a data management system, collecting relevant SWC-related data sources while ensuring privacy and security aspects. Such data are often unavailable. The collaboration with PoL enabled the access to a large dataset with a high time resolution, collected over several years in Luxembourg (2015–2019).

PoL is a waste collection company which collects waste of private clients (e.g., companies, restaurants, schools). Table 1 shows the difference between public and commercial waste collection. Please note that these differences were detected in Luxembourg and can differ in other countries. Still, the differences of both collection services are significant and can be further clarified by their modeling as operational research (OR) problems [9]. Public waste collection can be modeled as a variant of the ’chinese postman problem’, where the objective is to find the shortest path that visits every *edge* (street) of an undirected graph. Commercial waste collection can be modeled as a variant of the ’traveling salesman problem’, where the objective is to find the shortest path that visits every *node* (city, client) of an undirected graph exactly once [10].

The rest of the paper is organized as follows. Section 2 reviews the prior state of the art by highlighting the existing solutions. Section 3 describes the case study that was used, the opportunities in terms of business and environmental impact reduction, and a high-level architecture of the proposed SWAM platform. The data collection methodology that involves different datasets and details of each of them are presented in Section 4. Data analytics and discussions are highlighted in Section 5 and Section 6 respectively. Finally, future research directions are presented in Section 7.

## 2. Related Work

Waste management systems (including waste transportation, disposal, and recycling) can be categorized into three main types:

- Sensor-based waste collection (e.g., dynamic adaptation of trucks routes, and optimal planning and scheduling of waste collection trucks).
- Waste transport-based location intelligence (e.g., route optimization based on the waste type).
- Recycling solutions.

A large body of research on waste management have been proposed, and this section focuses on the first type (i.e., sensor-based waste collection). The usage of sensors for SWC was introduced several years ago using RFID chips [11]. Most recent studies used advanced IoT technologies such as automatic fill level sensors, cameras or laser in order to detect the bin fill-level. However, it is already highlighted that these technologies suffer from several issues (e.g., accuracy, sensor integration, and battery lifetime). As a result, this study aims to remedy these limitations by using ultrasonic filling detection sensors [12].

Table 2 displays a comprehensive analysis, conducted on the prior art on sensor-based waste collection, which was discussed in this literature. The commentary that follows describes the key achievements and milestones that have taken place.

The usage of weight and proximity sensors was introduced in [13] to continuously measuring the filling level and the amount of dump in each bin. The aim of the study was to optimize the routing operations. Nevertheless, the proposed study was based on simulation rather than real life tests.

A dynamic waste management model was proposed by [14]. The authors developed a platform, called CloudSWAM, that aims to automatically store the waste filling level of different bins types (i.e., organic, plastic and bottles, and metal) into the cloud and subsequently use this information to effectively and dynamically optimize the truck route direction, transport the waste based on the type and recycling.

Another study by [15] focused on identifying the best location for landfill based on GPS data, and the genetic algorithm approach was applied for selecting the optimal area for disposal of domestic and industrial waste.

A solar powered compacting bin was used in [16] in order to predict the bin fill-level and offer automatic compaction of waste, effectively increasing the bin capacity by up to 10 times. Data were transmitted into a cloud server and were used to optimize the waste collection route and offer dynamic scheduling plan.

The Smart Garbage Bin is a tool that was introduced by [17] in order to optimize plastic recycling and to predict the amount of waste in bins for a better collection decision by the city council. The authors used near-infrared reflectance spectroscopy in order to isolate the different types of plastic as some of them are not degradable and could result in land pollution while the capacity sensor was used to estimate the bin filling level. Nevertheless, the collected information was not effectively used to provide Dynamic Routing and scheduling.

To optimize or provide an indication to the requested number of bins for each client, real life evaluation was conducted in [18] to measure the bins filling level by using ultrasound sensors. However, the case study evaluating the system efficiency was very limited. The IoT sensors were deployed in 11 containers only.

Another study by [19] applied the genetic algorithm to analyze the collected bin information. The goal was to offer optimized path for waste collection. The experimental setup involved the usage of several technologies (i.e., ultrasonic sensor, Arduino micro-controller, GSM/GPRS shield, and buzzer) to predict the amount of waste inside a bin, which is costly to implement in real life scenario. Once data has been collected, a specified collection threshold value was set up. A notification containing the bin ID and location is sent when the filling level percentage meets or exceeds the predefined threshold value.

A dynamic routing algorithm was proposed in [20] to find the optimal path for two types of trucks (i.e., high and low capacity). The bin filling level was measured by using the capacity sensor while RFID was used to identify individual bin.

Another study was presented by [21] to minimize the waste collection cost and maximize the customer satisfaction. To sense the bin filling level using the capacity sensor, different sampling rates were tested (i.e., the fill level information was captured every one and two hours) and the automatic collection would be granted once the filling threshold reaches 70% or more. Moreover, the proposed solution recorded the significant changes during certain days to recommend the optimal day(/s) for waste collection and to find out the optimized routes. Nevertheless, the geographical distribution of the bins was rather limited and located in 10 regions only, which is limited when making an allegation of robustness.

For effective handling of waste collection for indoor containers (i.e., shared bins by multiple households), a case study by [22] was explored by using real life data obtained from the Twente Milieu, which is a waste collection company in the Netherlands. Since the stakeholders already performed the dynamic waste collection task based on the fill level information (that was captured from the equipped capacity sensor), the focus of the study was to define effective routes for each truck during collection using the heuristic approach.

The creation of waste type profiles was introduced in [23] by using the combination of k-means with the self-organizing map algorithms. The proposed approach used the container-level waste weighting information, GPS information of bins, bin size, and waste type in order to understand the relationship between consumer behavior and the produced waste. Extensive data collection was conducted by involving information of more than 185000 pickup trips over two years in Helsinki, Finland (2013–2015).

Table 2 shows that the majority of studies were usually designed for general public target markets and based on simulation experiments (apart from [22,23] that used real life test). Moreover, current solutions mostly rely on limited flow of information and thus result in limited decision-making. To this end, the objectives of the proposed system (i.e., SWAM) are to explore, investigate, and analyze multiple sources of information such as the bin filling level, truck GPS information and client profile in order to enhance the decision-making process as well as to focus on business services (rather than public waste management).

Our previous work [7] provides detailed information on the challenges and issues that must be addressed to overcome the aforementioned issues, which results in the creation of three complementary modules: data management, multi-objective optimization, decision-making component. The next section describes the methodology of our research studies, and focuses on the data management layer specifically.

## 3. Use-Case

### 3.1. Scenario

The authors of the present study are working together with PoL that offers a waste collection service for professionals and large organizations. This service has been operating for several years in Luxembourg, a small country but, nevertheless, an ideal place for testing and validating research hypotheses. This country has a road infrastructure that is representative of other European countries and its cross-border situation with France, Belgium and Germany generates an exceptionally large amount of daily road traffic. In this context, the SWAM project was developed to set up an advanced waste management platform composed of several modules. This paper focuses on the data management module.

**Figure 1:**
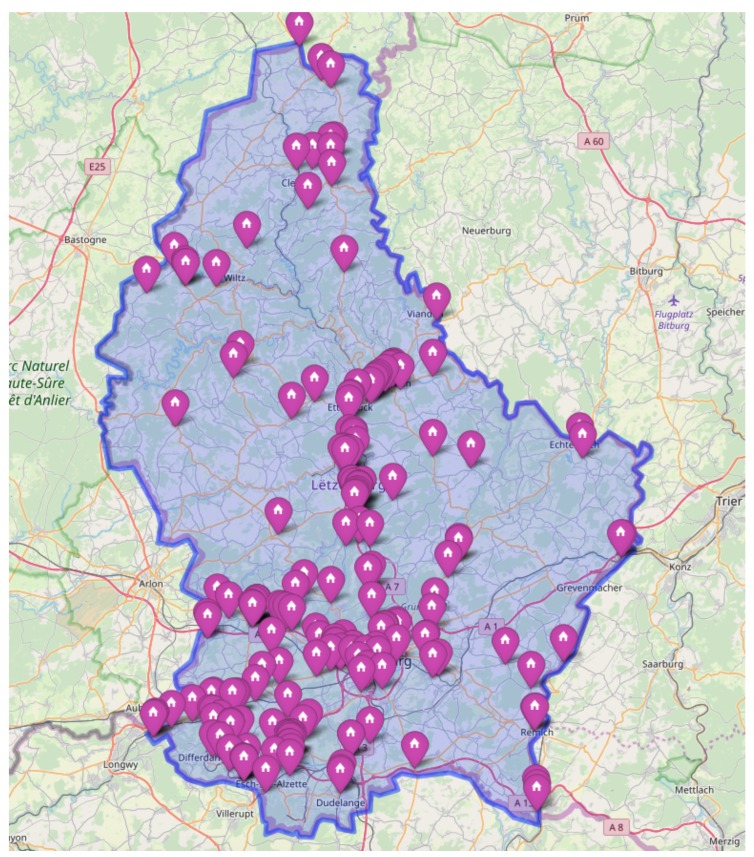
Clients’ spatial distribution on the complete country (Luxembourg) [7].

Figure 1 provides the spatial distribution of PoL’s clients, which covers the whole country. Poles of activity such as Luxembourg-City or Esch-sur-Alzette, the two main cities, are naturally represented. On the current system, customers are spatially clustered into two zones, i.e. the north and the south, so that drivers can easily share the collection work.

**Figure 2:**
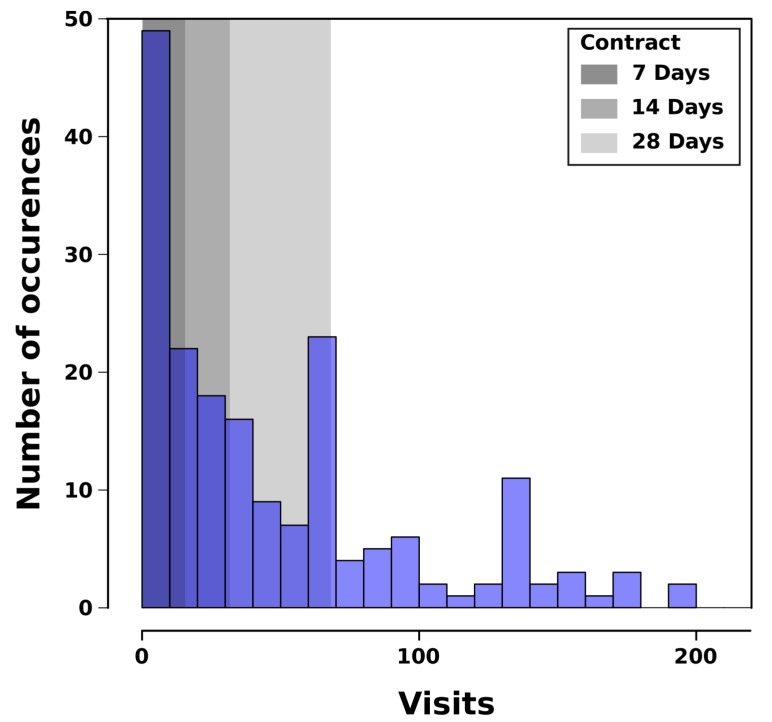
Distribution of the number of visits by clients in Luxembourg, from February 2018 to May 2019.

This service offers waste collections with a fixed frequency, depending on the contracts and the type of waste to be collected. The collection performance is thus entirely dependent on the driver and the very low level of information available to him. Unlike public waste collection for private individuals and municipalities, the visited companies do not have homogeneous behavior depending on their socio-professional activity (i.e. NACE code, in Luxembourg) and their level of activity. This is particularly visible in Figure 2. Depending on the type of contract, some customers can be collected weekly, monthly, or sometimes on demand via direct phone calls, which naturally generates a waste of time and does not optimize the use of PoL’s resources.

This study therefore considers this scenario, and looks at the different types of data that can be used to improve the situation and ultimately optimize the company’s decision-making processes. In particular, we deployed 47 sensors all around Luxembourg on domestic waste containers.

The following section highlights the expected impact of the SWAM solution.

### 3.2. Impacts

**Figure 3:**
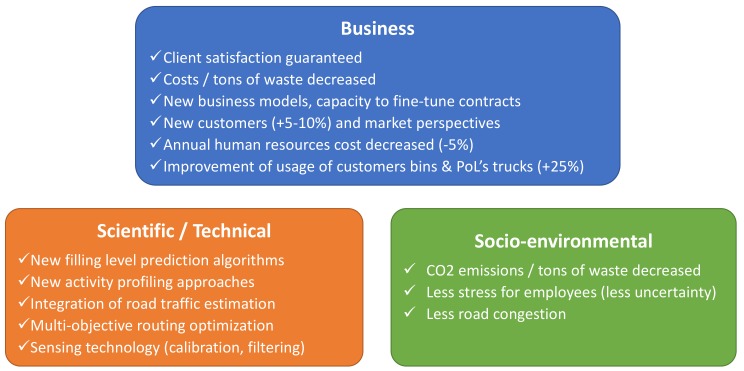
Impacts generated by SWAM.

Through SWAM, three main categories of impact are foreseen and are summarized in Figure 3 below. These impacts have of course to be assessed over various periods of time. For example, it is obvious that socio-environmental impacts can only be assessed over the long term, whereas scientific, technical and business impacts can be assessed progressively once the technologies described in this paper are in place. More details can be found in [7].

The next section provides a view of the SWAM architecture.

### 3.3. Architecture and Parameters of the System

**Figure 4:**
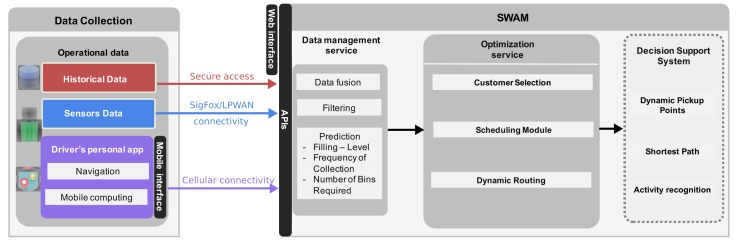
SWAM—High-level architecture.

A high-level architecture of SWAM is presented in Figure 4.

The SWAM architecture consists of four main core components:

- **Data Collection:** it includes different datasets that are captured from several resources. This involves the historical data from the SWC company, namely ERP data while the sensor data are collected with ultrasonic sensors deployed around Luxembourg. Finally, the vehicle tracking information would be used to identify its location in nearly real-time. A relational database is used to store all the collected data (SQL server). Details of each dataset are described in Section 4.
- **Data management service:** managing varying data resources becomes quickly complex. After the completion of the collection phase, a filtering process would be conducted out to explore the accuracy of the generated information. Predictive models will handle missing fill-level data. Regression mechanisms will enable prediction of the filling level at least 48 hours in advance.
- **Optimization service:** it is mainly divided into three parts:
  - **Customer selection:** the relevant customers are selected for an inclusion into a waste collection, by considering a set of objectives and constraints.
  - **Dynamic Scheduling:** this module is designed to offer optimal planning and waste collection scheduling (i.e., waste collection would be executed only if it is needed).
  - **Dynamic Routing:** the selected customers are integrated into a fleet management optimization engine that generates a dynamic adaptation of trucks’ routes and selects the optimal path.
- **Decision Support System:** this interface involves three main modules:
  - **Dynamic Pickup Points:** this module aims to generate automatic pickup points that should be considered by the driver.
  - **Shortest Path:** this module is responsible on recommending an optimal path based on the current vehicle location and the ordered list of clients to be served.
  - **Activity Recognition:** unlike most of the prior studies that have not considered the driver behavior, the proposed system aims to use smartphones and use the embedded motion sensor in order to capture the driver’s activities. Detecting what a driver is doing at a specific point of time would enable the creation of dynamic profiling for each driver independently. For instance, calculating the service time of each collection per site could give an indication of the actual number of processed bins.

The datasets used in our research studies are presented in the following section.

## 4. Datasets

Many metrics are collected after the completion of a waste collection in each client site, i.e., site id, client id, client NACE id (i.e., socio-professional category), collection date, collected weight for each emptying bin within the truck which is equipped with a weight gauge, type of waste, etc. We present in this section the available datasets that enter into consideration. Section 4.1 considers the information extracted from PoL’s ERP. During its daily operations, PoL stores a lot of data that were not analyzed until our research study. Our aim is to apply time series analysis on this unused knowledge in order to profile the weight production of each PoL’s client. In Section 4.2, we present another dataset available and extracted from GPS trackers mounted on each PoL’s truck. These data will be used to characterize the service duration (waste collection) in each client’s site. Finally, Section 4.3 describes a new source of information that will be generated after the deployment of ultrasonic sensors in PoL’s bins and that enable a remote monitoring of their fill level. The goal is to be able to plan the right waste collection timing based on real fill level information.

### 4.1. ERP: DIVALTO

DIVALTO [24] is a management software providing a complete ERP solution. Its databases enable storing many parameters linked to daily waste activities. It is based on a SQL server. PoL provided a complete export of its database. Here is a non-exhausted list of available features available for each activity:

**Client** 
  - ClientName/ClientNameId refers to the client’s name in a human readable-format and its unique identifier in the ERP database. Each client may have multiple sites where bins are actually located.
  - ClientAddress is composed by the street name, the city name and the zip code in a human readable-format.
  - ClientActivity/ClientActivityId corresponds to the client’s activity (Retail, Construction, Housing, etc.) in a human readable-format and its unique identifier in respect with the European regulation (NACE code) in the ERP database.
**Site** 
  - SiteName/SiteNameId refers to the site’s name in a human readable-format and its unique identifier in the ERP database.
  - SiteAddress is composed by the street name, the city name and the zip code in a human readable-format.
  - SiteObservation/SitePreference provides any site information (key, phone number, etc.) and service preference such as the preferred time window (starting/ending time in the morning or in the afternoon, day or time-slot when collection is prohibited, etc.).
**Activity** 
  - Each activity (bin emptying, bin exchange from an initial volume to a new one, etc.) is defined by a unique code in the ERP database, and that for each stage of the execution (from the command, to the intervention on site and finally when the service is done).
  - WasteType provides the type of waste in a human readable-format that is planned to be collected. It enables filtering waste collection activities (e.g., domestic, glass or cardboard).
  - WasteVolume refers to the bin volume (maximal capacity).
  - WasteWeight provides the collected weight.
  - WasteTimestamps corresponds to the service date.

These parameters are updated into the ERP after each bin emptying. Thus a new entry is generated into the database, with the associated weight recorded from the truck weight sensor. For instance, if 4 bins are available on a site but if only 3 bins are full and finally processed, 3 new entries will be added. The bins are rented by PoL and their quantity on each site are negotiated, based on the clients’ needs, i.e., estimated waste production, available waste fraction on site such as domestic waste or glass, indoor/outdoor location, collection frequency (7 days, 14 days, 28 days, or on demand with phone calls), etc.

**Figure 5:**
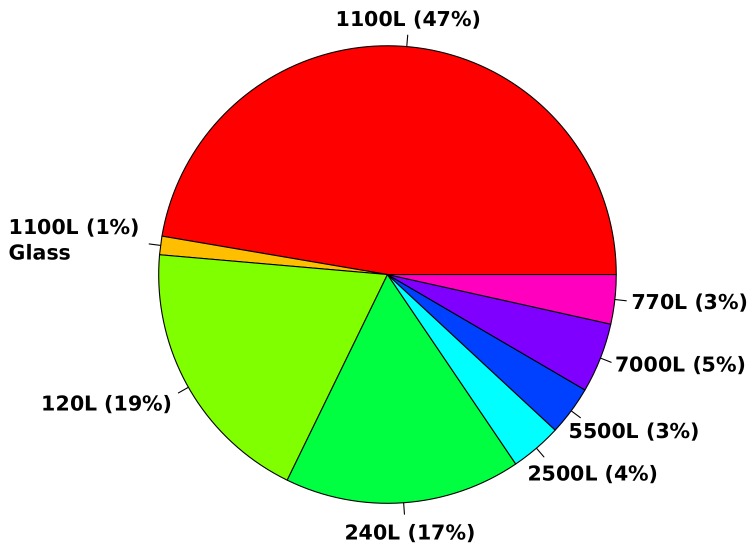
Bin volume distribution (domestic waste/paper-cardboard and glass) for waste collection processed in 2018.

Different volumes are proposed by PoL, ranging from 120 L to 7000 L. Figure 5 highlights the variability of the containers for all processed bins related to domestic waste, paper-cardboard and glass collected in 2018. In fact, each waste fraction is placed in a separated bin. Thus, PoL’s driver collects a defined type of waste during his working day and then empties his truck in a sorting center. As a matter of course, the content has to be homogeneous. If the content is mixed, PoL has to pay extra-fees to the sorting center. As a consequence, each driver operates a unique fraction of waste (for instance domestic waste) during his working day, and then empties the collected waste in the sorting center. Finally, the truck is cleaned and is ready for the next collection.

The truck location is monitored in respect with a tracking solution that is presented in the next section.

### 4.2. WinFleet

WinFleet is a software solution for fleet management developed by SkyCom [25]. Companies can use this software to monitor the current location of their vehicles, to analyze the paths traveled by their vehicles and to generate summarizing reports. WinFleet is typically used via its graphical web-interface; for further processing it is possible to export and download the data in CSV-format. This dataset presents the following information for each entry:

**Time**  The timestamp of the entry.
**Status**  Information on the individual truck’s state:
  - Is the motor running?
  - Is the truck stopped or is it driving?
  - How fast is the truck moving?
**Position**  GPS-information where this entry was taken:
  - Latitude.
  - Longitude.
  - Street name in a human readable-format.

In our use-case, this dataset holds all information about the individual waste collection truck’s position over time. With this data, it is possible to reconstruct the trucks’ collection path, and extract new meaningful information. Possible examples for new information could be (a) the influence of traffic density on travel duration or (b) the service duration at a client site depending on the time and the weekday.

The extraction of new and meaningful information out of these data sets is not a trivial task. One has to clearly identify and assign the different stops of a truck. Did the truck stop because of a traffic light, the lunch break of the truck’s driver, or a waste collection at a client’s site? Furthermore, the collection tours can present detours which can be difficult to identify and explain. Did the driver adapt his path because of the traffic density, a road block or a car crash? The exploration and analysis of WinFleet data can be found in Section 5.2.

The last data resource is generated by the sensors deployed in Luxembourg in order to retrieve bin fill levels. It is described in the following section.

### 4.3. Sensors

**Figure 6:**
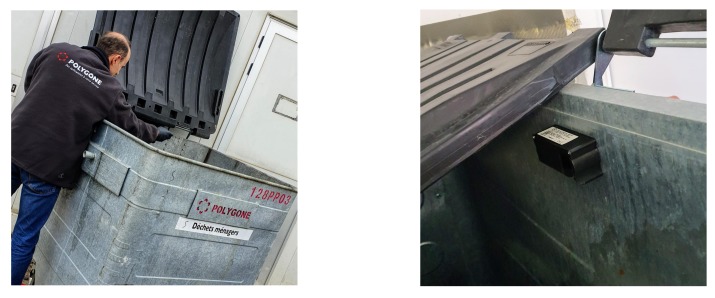
Deployment of one sensor into a container.

The system we present in this study relies on ultrasonic sensors deployed into waste bins all around Luxembourg. At the date of writing this paper, 47 sensors have already been deployed on 35 different sites. Figure 6 shows an example of sensor deployment. In 2020, it is planned to reach 200 units, and in the longer term to cover all the customers of PoL. This section describes the main technologies that integrate this sensing component.

#### 4.3.1. Ultrasonic Sensing

**Figure 7:**
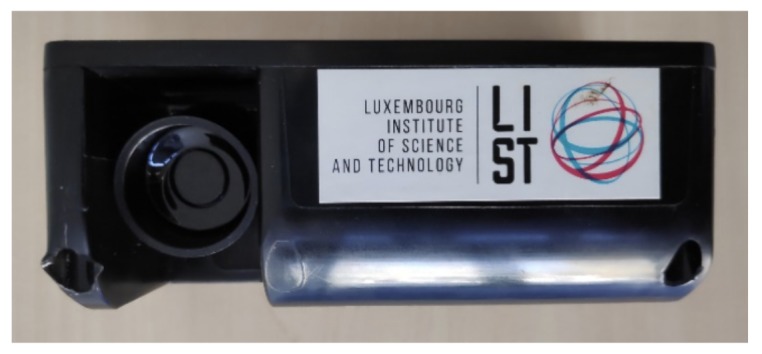
Brighterbins ultrasonic sensor (L=130mm, W=70mm, H=53mm).

Brighterbins’ ultrasonic sensors [26] (illustrated in Figure 7 were selected among 41 potential suppliers. This choice is based on many factors such as price per sensor unit, software/hardware independence, communication technology, system lifetime, integration ease, waterproofing, robustness and measurement accuracy. The full list is available in [7].

**Figure 8:**
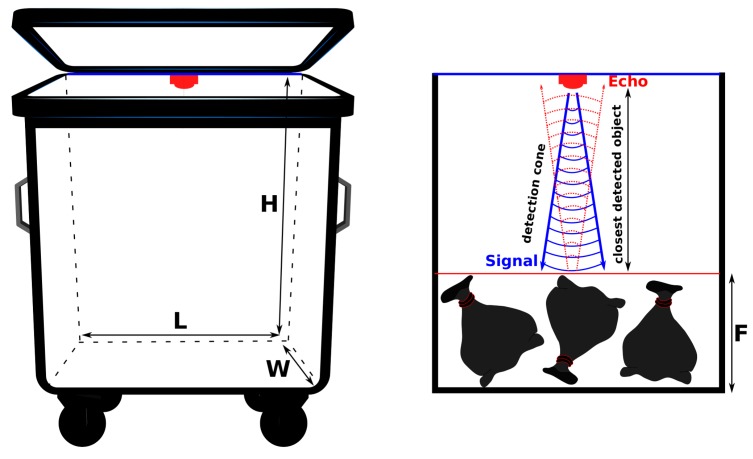
Sensor integration under the hinge in a container.

Figure 8 describes the distance measurement process. An ultrasonic sensor sends a sound wave (in blue) at a defined frequency, and waits for the echo (in red) generated after its reflection on an obstacle. The distance to the closest obstacle is determined by measuring the time laps between the sent and received ultrasonic pulses. The time is then converted into distance in respect with the speed of sound (340 m/s). F represents the fill level (distance between the bottom of the bin and the average waste plan). By definition, F≤H where L, W and H are respectively the bin’s length, width and height. The volume of garbage bags can vary from small (40 L) to extra large (240 L) and can be partially filled with waste. As a consequence, the average fill level has to be estimated. Each bin is assumed to be homogeneously filled. Thus, the sensor measurement is assigned to the average fill level. The sensor integration is described in the next section.

#### 4.3.2. Sensor Integration

**Figure 9:**
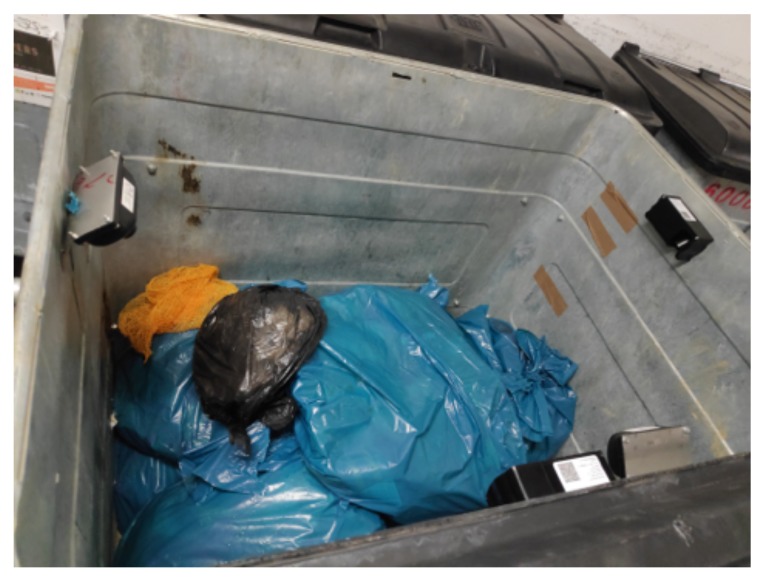
Testing of 4 sensors deployed into a container.

Setting up a sensor into a container is not an easy task, since we need to consider the type and size of container, the inclination angle, the communication technology constraints, and a few other parameters in order to ensure the best possible quality of measurement. In addition, the use-case considered in this study is very specific and consider professional containers, which are bigger than the conventional public bins and with no or limited possibility to deploy the sensors under the lid (i.e., it is build with fragile material). The impacts between a sensor and waste bags during their emptying within the truck should also be considered. Therefore, as shown in Figure 9, we deployed different sensor models at different locations and measured their reliability.

Based on this initial deployment, the most appropriate location for the sensors was decided to be just below the lid, under the hinge (see Figure 8). The network communication is presented in the following section.

#### 4.3.3. Network Communication

We considered different communication technologies to retrieve measurements from the sensors. Cellular technologies were first considered, since they are well deployed over the country and have an excellent quality of service. However, a SIM card has to be inserted inside each sensor during the network set up. This manual operation becomes unmanageable for a large deployment. Moreover, energy consumption is too high compared to other technologies.

In the proposed scenario, SigFox is preferred and satisfies the technical requirements. Sigfox belongs to the Low Power Wide Area Network (LPWAN) family, in the same way as LoRA or NB-IoT [27]. It is already deployed and provides a full coverage of Luxembourg, with a good level of redundancy (at least 2 or 3 antennas for most of the deployment sites).

**Figure 10:**
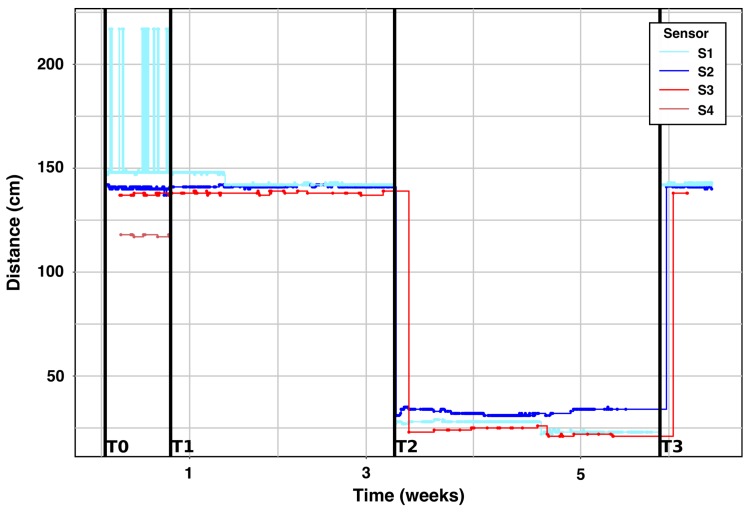
Data collection of 4 sensors placed into a 1100 L test bin [7].

Fill-level time series for the integration described in Figure 9 (4 sensors) are shown in Figure 10 for a duration of 6 weeks starting from 24 April 2019. This set up was designed in order to evaluate the best sensor location enabling accurate measurements. S1 and S2 are sensors from Brigtherbins (Sigfox class 0), S3 and S4 from Sensoneo (Sigfox class 2—made for outdoor environments, at that time).

**Figure 11:**
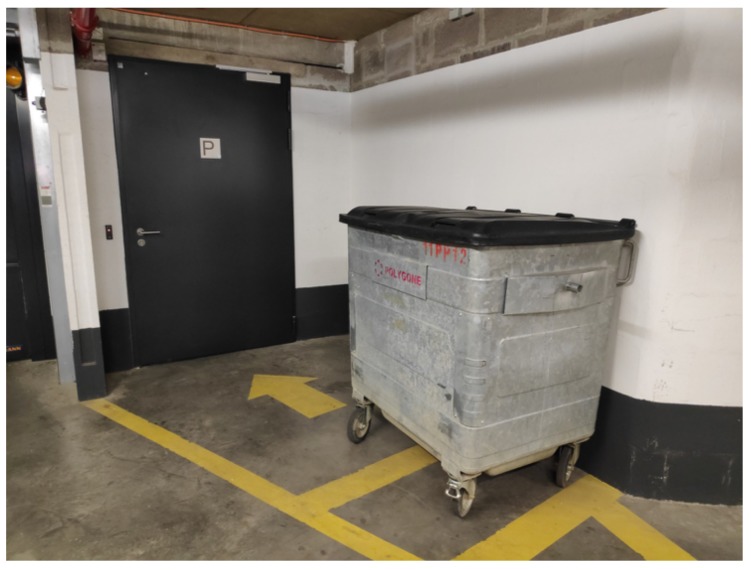
Indoor technical room where Sigfox connectivity was tested.

The time T0 indicates that the bin moved to the entrance of the client’s building. At T1, the bin was placed in front of the technical room (indoor, see Figure 11). At T2, an activity is detected, i.e., the fill level is quickly changing from an empty state (Distance = 135 cm) to almost a full configuration. Finally, at T3, a waste collection occurred, and the fill-level comes back to its original value.

S1 showed very unstable measurements during the first week, explained by multiple reflections of the ultrasonic beam. This issue has to be handled during the data cleaning. However, Brighterbins’ sensors were overall more stable from a communication point of view.

The recorded metrics are presented in the following section.

#### 4.3.4. Metrics

The sensors we installed are configured to send the following information a few times a day (minimum twice).

**Time**  The timestamp of the entry.
**Distance**  The distance (in cm) from the sensor to the nearest obstacle. This makes it possible to deduce:
  - The filling level of the bin (depending on the dimension of bin).
  - Alerts based on critical fill levels.
**Temperature**  The temperature of the bin, in degrees Celsius, for:
  - Fire detection.
  - Weather information retrieval.
**Battery**  The power level of the sensor, which are designed with a 5-year, interchangeable battery (according to the manufacturer’s information).

The next section focuses on analytics computed from these 3 datasets.

## 5. Data Analytics

We processed and extracted data analytics from each information source presented in the previous sections, i.e., ERP (described in Section 4.1) in Section 5.1, Winfleet (highlighted in Section 4.2) in Section 5.2, and finally ultrasonic sensors (presented in Section 4.3) in Section 5.3.

### 5.1. ERP: DIVALTO

PoL has updated its ERP in January 2019 to the DIVALTO solution (see Section 4.1). The previous database has not been included into DIVALTO. In fact, many modifications were done to simplify the previous system (new identifiers, codes, fields, etc.) and the inclusion of the previous dataset into DIVALTO has become unmanageable. However, we decided to use the previous dataset (more information on a larger time scale) in order to perform a deep analysis of clients’ waste production, i.e., profiling. During data cleaning, we discovered some errors such as wrong weight insertion (WasteWeight). As a reminder, the weight is measured during the bin emptying and printed into a ticket by a device located into the truck cabin. When the waste collection is finished, all tickets (40 served clients on average) are currently transmitted by the truck driver to the PoL’s dispatching department which thereafter has to include them manually into the ERP. This encoding may add some errors. An automatic transmission is not available now, but may solve this encoding issue. We also found in WasteType many names designing the same type of waste. Aggregation was done in order to extract all related entries focusing on a defined fraction of waste (i.e., domestic waste) for all possible contracts (7 days, 14 days, 28 days, and on demand).

**Figure 12:**
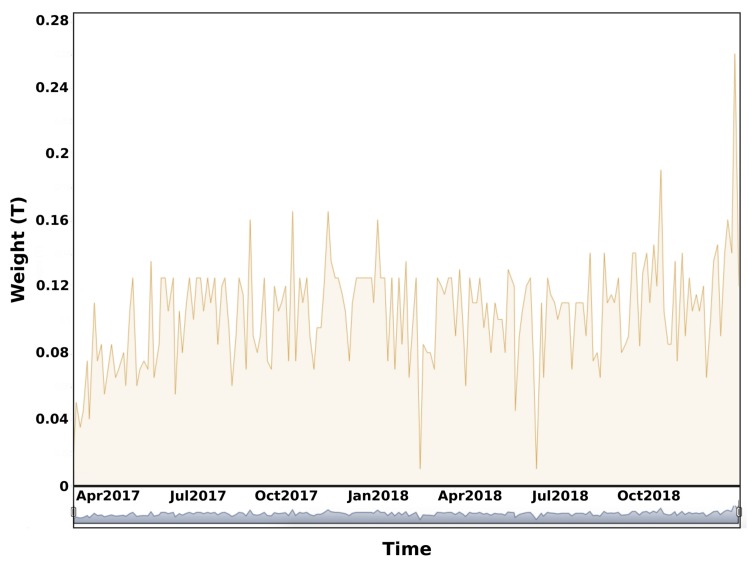
Example of the collected weight time-series extracted for a selected retail business.

The time series associated with a retail business is presented in Figure 12 after data cleaning. We observe a high variability that can be explained by many reasons. The waste production of a client may change according to new activities or events. A second explanation relies on synchronization issues between the waste collection tour (PoL) for a fixed contract (e.g., one collection every Monday) and the real waste production.

To compare and analyze the impact of the business activity (i.e., retail business, construction, housing activity and restaurant) on the weight production, we propose to compute on PoL’s domestic waste datasets composed by 112 clients, the following set of features (29 variables), i.e., (minimum, mean, median, standard deviation, maximum) weight, time-series length and duration in days for normalization, (minimum, mean, median, standard deviation, maximum) bin volume (changes can occur, i.g., exchange from 1100 L to 660 L), (minimum, mean, median, standard deviation, maximum) density (weight collected/maximal capacity on site), weight integral on the complete time-series, (minimum, mean, median, standard deviation, maximum) weight peak, number of peaks and (minimum, mean, median, standard deviation, maximum) time interval between successive peaks.

As each PoL’s client belongs to one of the 4 distinct activities presented earlier, we propose to apply the k-means algorithm in order to analyse the correlation between each activity and the associated waste production. k-means is one of the most commonly used unsupervised machine learning algorithm to separate a dataset composed by elements into k clusters represented by their gravity center [28]. Hartigan and Wong defined the standard algorithm where the distance between each component of a cluster and its center is minimized. Elements from the same cluster (respectively different clusters) present a high (respectively low) similarity. In our scenario, k=4. Unfortunately, k-means is very sensitive to outliers. Partitioning Around Medoids (PAM) [29] is less sensitive to noise and outliers than k-means. It is based on medoids. A medoid refers to an element of a cluster for which average dissimilarity between it and all the other cluster’s components is minimal. In PAM, medoids are used as cluster center instead of gravity center (means). The PAM algorithm is based on the search for k medoids among the dataset’s observations.

A scatter plot based on 2 selected features can be used to visualize the correctness of each presented clustering algorithm. We used on the one hand the weight median that is connected to the production of waste and on the other hand the weight standard deviation that shows the weight variability for all successive collections.

**Figure 13:**
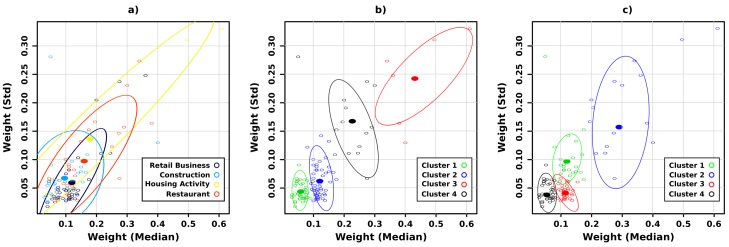
(**a**) Dispersion plot: weight median vs weight standard deviation. (**b**) A clustering k-mean algorithm was applied for k=4. (**c**) A clustering PAM algorithm was applied for k=4.

Figure 13 a shows the dispersion plot for the previous metrics. Each client is represented by a circle whose color is related to the client’s activity. We displayed the confidence ellipse (80%) for each activity group. We observe a high overlapping between all groups. Unfortunately, we can conclude that the two selected metrics (weight median and weight standard deviation) are not sufficient to classify each group member into a unique cluster. In fact, our goal is to be able to classify each client waste profile in respect with its activity.

The application of the k-means clustering algorithm was applied and the result is shown in Figure 13b. Each point represents a client and is displayed with a color associated with its cluster assignment. We can see that the generated clusters are well separated according to metrics’ values but there is no general correlation between the computed cluster id and the real activity. Table 3 gives a summary of their correlation. It provides the correlation matrix between the k-means clustering and the real activity of each client.

Let’s consider the population of retail businesses R (black circle in Figure 13a). 30.2% of R are classified into the cluster 1, 58.1% into the cluster 2, 2.3% into the cluster 3 and 9.3% into the cluster 4. We observe that the four activities are mainly classified into the clusters 1 and 2. As a consequence, a k-mean algorithm applied on weight median and weight standard deviation does not enable us to separate each population from each others.

The application of the PAM clustering algorithm was applied and the result is shown in Figure 13c). The generated clusters are also well disjoint but there is no general correlation between the computed cluster id and the real activity. Table 4 presents the correlation matrix between the PAM clustering and the real activity of each client.

To detect which features will enable a correct classification, we propose to apply Principal Component Analysis (PCA) on our feature matrix (112 observations with 29 variables) and display each observation according to its first two principal components. In fact, feature extraction is a key component to design an effective system [30], therefore, a comprehensive feature extraction process was carried out. PCA were used in order to identify the most distinctive feature subset. The feature selection step has become the focus of many research studies in order to reduce potentially large dimensionality of input data and thus system performance could be enhanced by selecting the most optimal and unique features [31,32].

**Figure 14:**
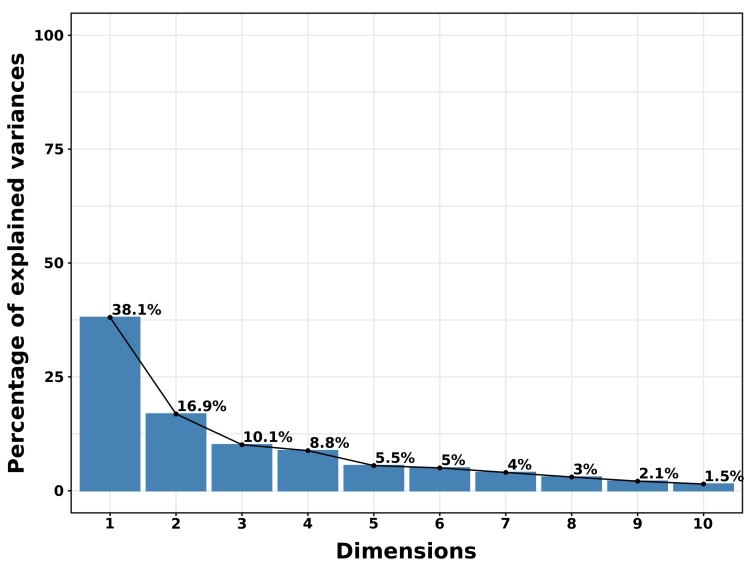
Variance related to eigen values.

PCA’s eigen values measure the quantity of variance explained by each principal component (see Figure 14). Eigen values are large for the first components that provides the directions related to the maximal dataset variation.

**Figure 15:**
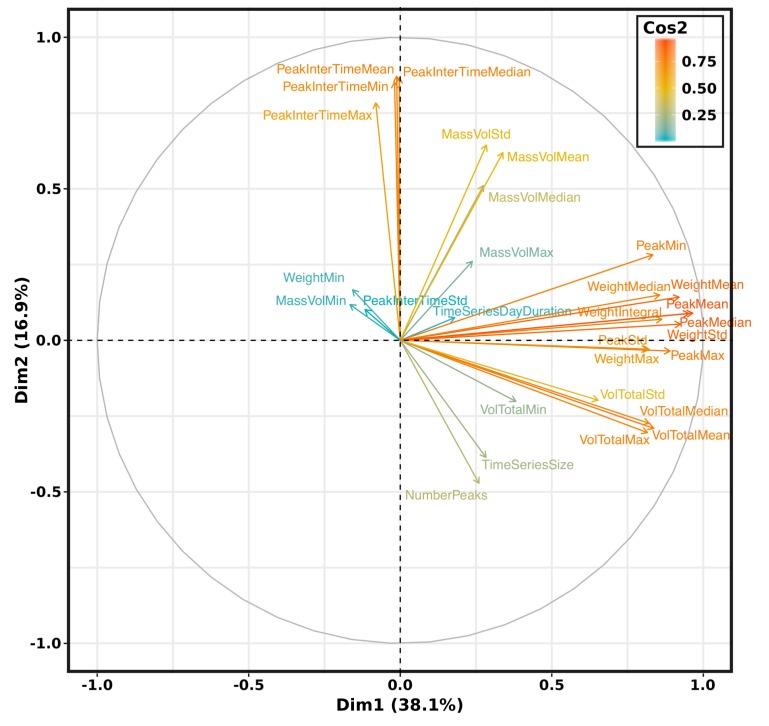
Correlation graph.

**Figure 16:**
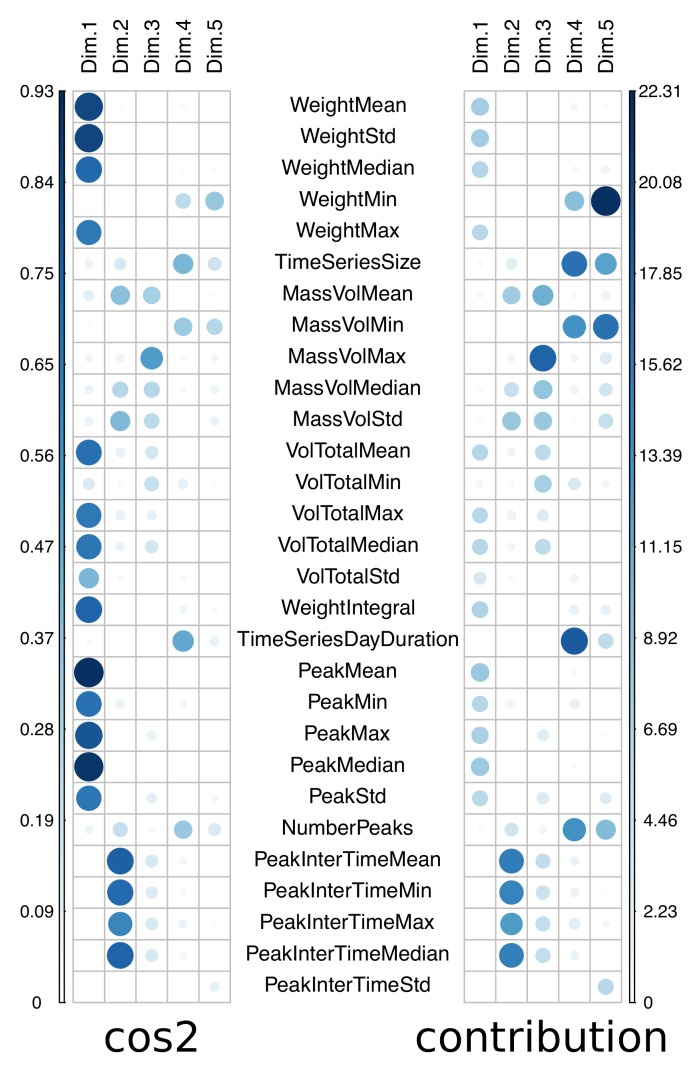
Correlation plot between features and principal components (cos2 and contribution).

The cumulative variance is 65% for the first 3 eigen values, respectively 73% for 4, 79% for 5, 84% for 6, 88% for 7, 91% for 8, 93% for 9). The first 7 components on the 29 provide almost 90% of the information. Let’s visualize the correlation between each variable and principal component (see Figure 15 and Figure 16).

The variables positively correlated are grouped (see Figure 15) in the coordinate system based on the first two principal components (55% of explained variance). For instance, the number of weight peaks (NumberPeaks) and the time series size (TimeSeriesSize) present a high correlation. The distance between variables and the origin provides the quality of the variable representation (cos2). The contribution of each feature in a principal component is also computed and displayed in Figure 16. For instance, the first principal component (Dim. 1) is mainly composed by 4 groups of features, i.e., the first one is composed by WeightMean, WeightStd and WeightMedian, the second one by VolTotalMean, VolTotalMax, VolTotalMedian, the third one by PeakMean, PeakMin, PeakMax, PeakMedian, PeakStd, and the fourth by WeightIntegral. The second principal component is mainly based on temporal data such as PeakInterTimeMean, PeakInterTimeMin, PeakInterTimeMax and PeakInterTimeMedian. The third principal component relies mainly on MassVolMax. TimeSeriesSize, MassVolMin, TimeSeriesDayDuration and NumberPeaks are contributing to Dim. 4. The fifth principal component is made of WeightMin, TimeSeriesSize, MassVolMin and NumberPeaks.

**Figure 17:**
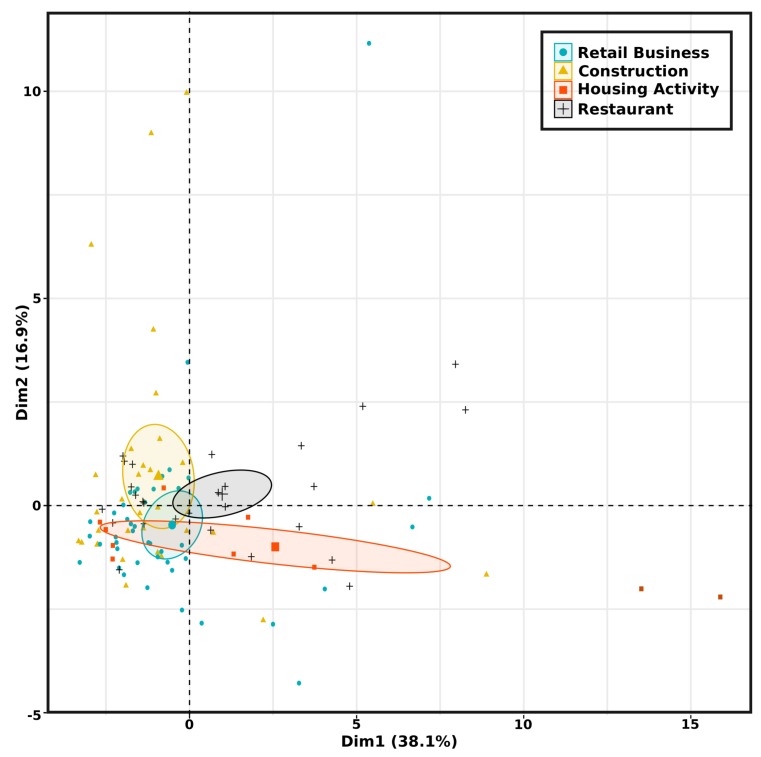
Dispersion plot in the PCA’s coordinate system (Dim.1,Dim.2).

Figure 17 corresponds to a new visualization of the dispersion plot (see Figure 13) in the coordinate system (Dim. 1, Dim. 2).

The clients’ classification based on their activity becomes easier because clusters are more disjoint in the PCA first dimensions. The proposed feature selection approach successfully maximized the classification accuracy with a reduction of nearly 75% of the whole features (i.e., only seven features were selected).

The correlation between each client’s activity and its waste production is not trivial and needs a deeper analysis. Additionnal factors may enter into consideration such as the company size, its location, etc. Moreover, the time granularity of each time series is fixed by each client contract (7, 14, 28 days or on demand). Then no information is provided between successive collections on each site. The use of sensors will fill this information gap (see Section 5.3).

The processing of Winfleet is presented in the following section.

### 5.2. Winfleet

**Figure 18:**
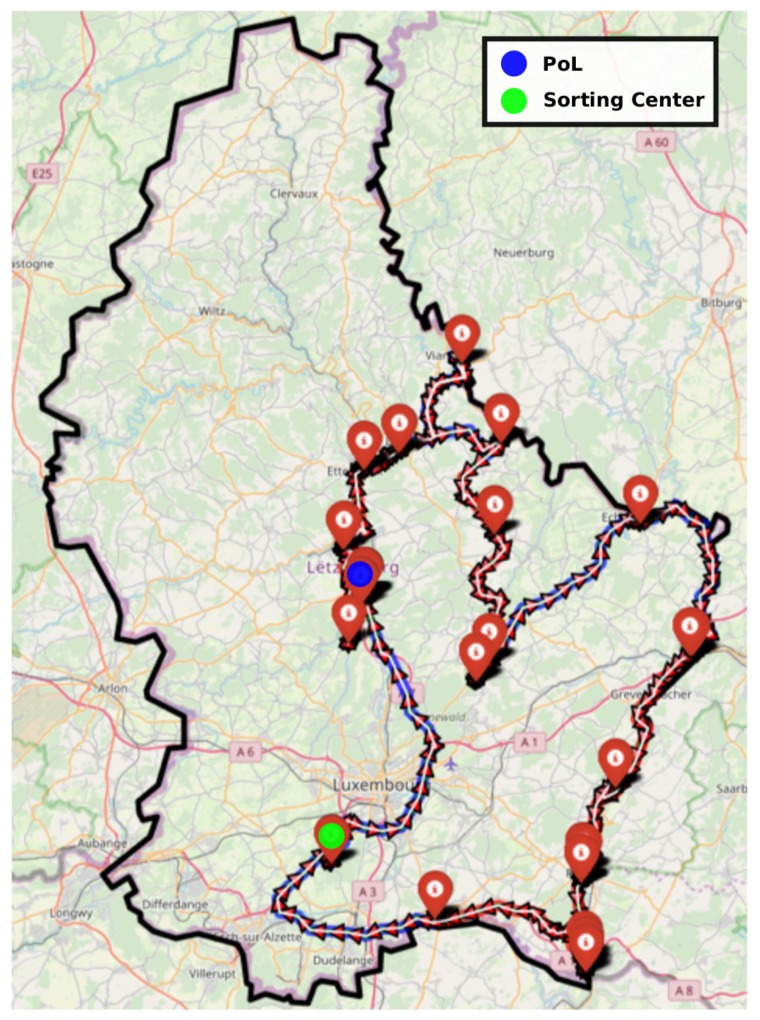
Example of a waste collection tour of a complete working day.

We designed python scripts in order to clean data exported with Winfleet (see Section 4.2). Figure 18 is the visualization of a waste collection tour done in the South of Luxembourg during a complete working day.

We can observe that the truck starts and finished in the PoL’s site (depot represented with a blue point). Each truck stop is displayed with a red marker. The driving path is displayed with colored arrows (blue and red). Each driving hour is represented with a single color. The transition from each 1-h time slot to the next one is done according to a color modification i.e., Red to Blue and so on. When the truck is full, it needs to empty itself in a sorting center represented with a green point in Figure 18.

**Figure 19:**
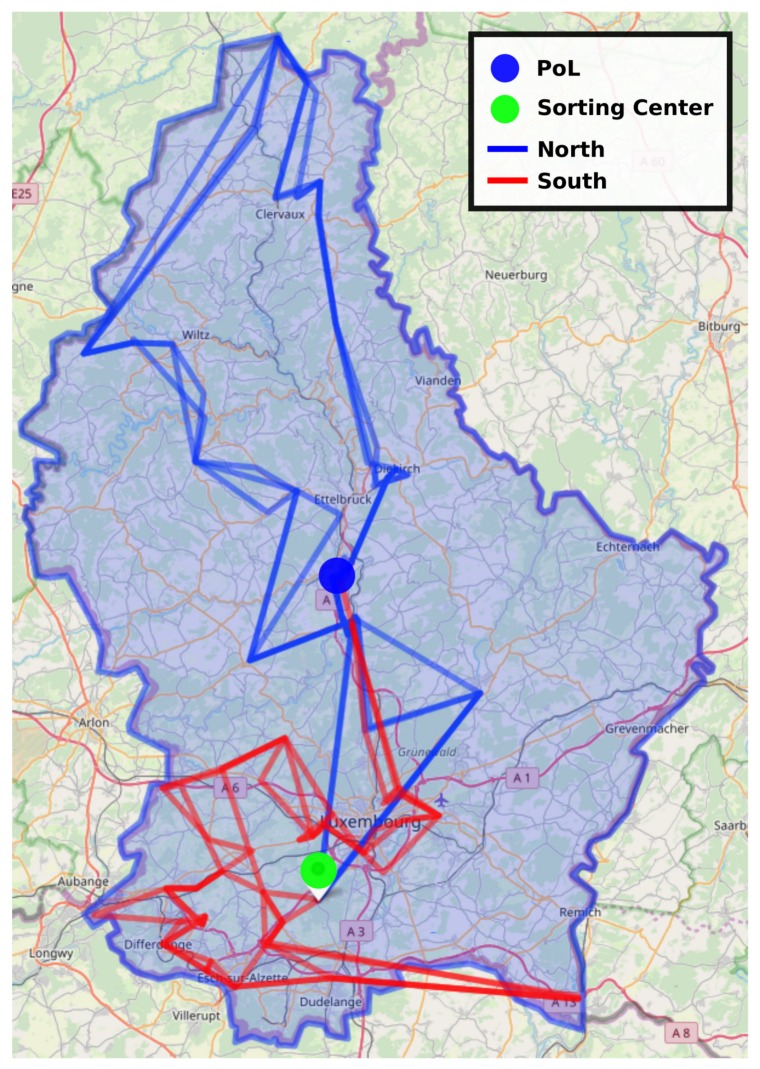
Successive collection paths on Monday (February 2019) for north and south tours.

Figure 19 shows the variability of the collection path for successive Monday in February 2019. We can distinct some changes (addition or removal of clients).

All stops have to be filtered. In fact, we have to consider only stops related to a waste collection, i.e., a site location. Thus, the truck location has to be close enough to at least one PoL’s client site whose address is provided by DIVALTO (see Section 4.1). The address of all sites was translated into GPS coordinate (Latitude, Longitude) in respect with geocoding tools such as geocoder graph hopper [33], or Google Maps APIs or the Geocoder python library [34]. In fact, many stops are due to traffic lights and road signs, traffic jams, working area, or drivers’ lunch break. We designed an automatic script to detect only stops related to a real service in any clients’ sites. The Euclidean distance between a stop under consideration and the list of potential clients/sites has to be smaller than a defined threshold (by default 20m). In fact, each bin can be placed outside the client’s building. So there can be a little difference between the real GPS coordinate where a bin is located and the site’s address. Moreover they are many configurations where the truck has to stop far from the client’s site, for instance just behind a barrier outside a building. In that case, the driver has to bring the bins from their locations to the truck in order to empty them. This action will add some service delays, especially if many bins have to be emptied. Sometimes confusion can occur, for instance when a stop is approximately at the same distance from two clients. These cases were rejected from our results.

**Figure 20:**
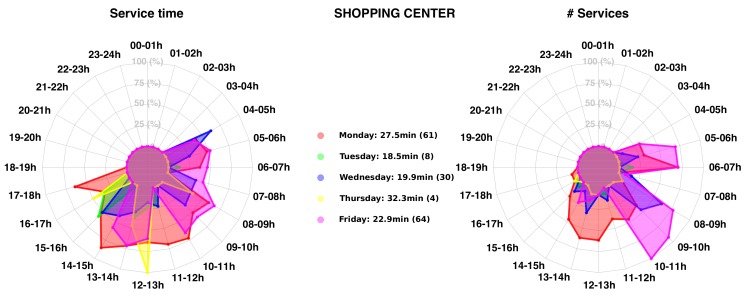
Service time profile for a selected shopping center.

**Figure 21:**
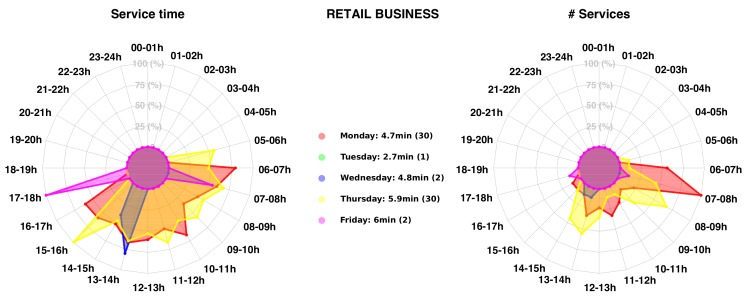
Service time profile for a selected retail business.

Then we computed many additional metrics related to the client profiling, such as the service duration D in second and the arrival time A of the truck on site. These new metrics were used to generate service profile for each site (see Figure 20 for a shopping center and Figure 21 for a retail business).

We extracted values (A,T) for all detected client/site stops from February 2018 to November 2019. For each 1-H time-slot, we first detected each waste collection (arrival and departure from the site location) starting within this time slot, and we computed the associated service time. Then, the average service time for all collections within each 1-H time slot was performed and displayed on a spider graph for each working day (duration and number of visits). The maximal radius (100%) represents the maximal service time and number of collections in any 1H-time slot. Other average service times are displayed proportionally to their values. For instance, for the shopping center represented in Figure 20, the waste collection on Monday has an average execution time of 27.5 min. This mean was computed on a set of 61 services. If the truck arrives on the site between 14–15 h detection (respectively 08–09 h), an average service time of 29.8 min (respectively 22.7 min) is reached. This spider chart enables finding the best time slot for an optimal waste collection on any clients’ site. The service time depends of course on the number of bins on site that has to be collected but also on the client activities and road congestion impacting the actual traveling time. For instance, for the shopping center, many other trucks are also delivering goods. That means that if PoL’s truck arrives on the site, it needs to wait that the delivery platform becomes free in order to empty the bins. This obviously adds delays on the process.

**Figure 22:**
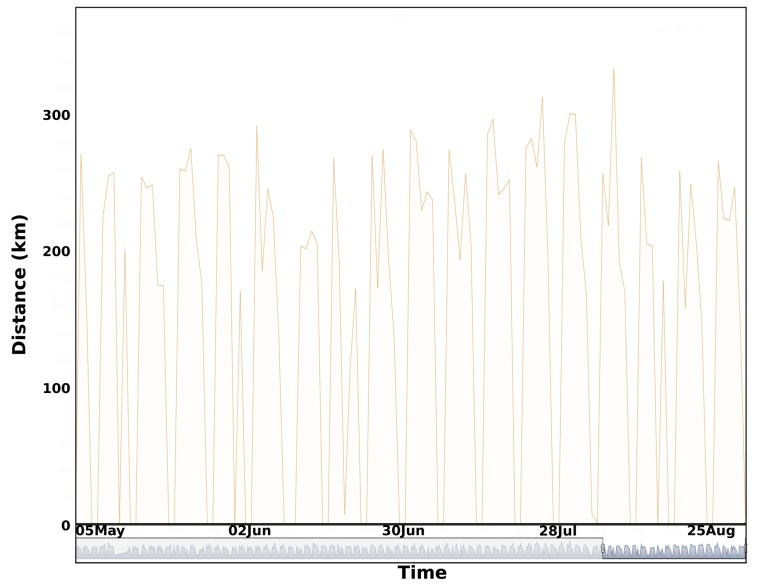
Traveled distance for the south waste collection tour between January–September 2019 (Average: 220 km, standard deviation: 54 km).

**Figure 23:**
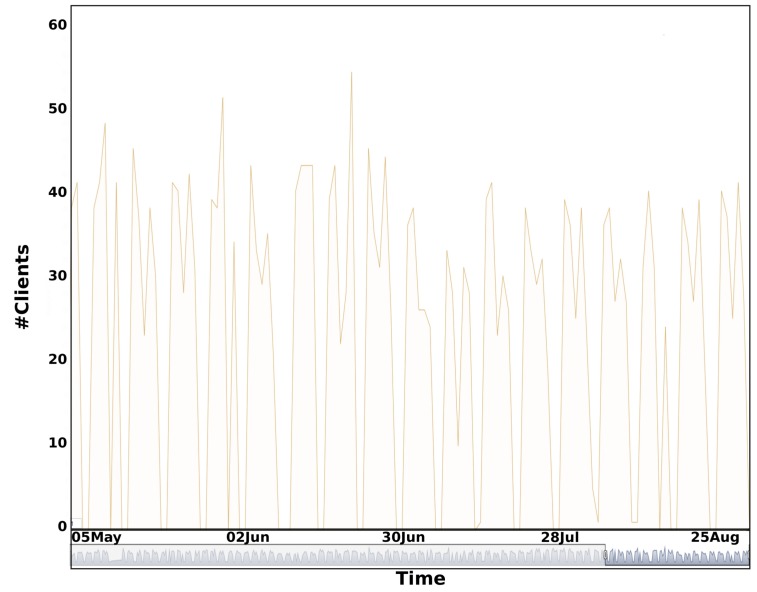
Number of clients served for the south waste collection tour between January-September 2019 (Average: 34 clients, standard deviation: 7 clients).

We also computed the total distance traveled during each complete tour for every working day and the associated number of clients in Figure 22 and Figure 23. We observe a high variability of the two time series. We can easily distinguish working days and weekends.

**Figure 24:**
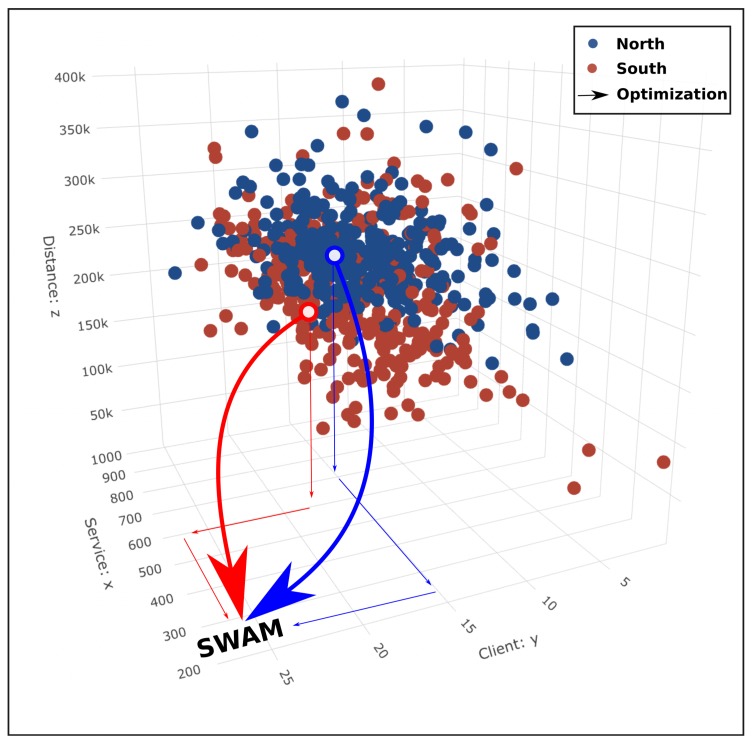
Dispersion plot (Distance, Client, Service time) for each tour (North and South).

Finally, we performed the average service time spent in each client site (in second), the total traveled distance (m) and the number of served clients for each tour (North and South). The 3D dispersion plot is shown in Figure 24.

The main goal of SWAM is to optimize the waste collection process by minimizing the total traveled distance and the service time on site according to each client’s profile and also maximizing the number of clients obtaining a service, and the collected weight.

The analysis of Winfleet data has enabled computing the average service time per site from GPS information. This metric will be included into the SWAM optimization module in order to schedule the best waste collection tour. We highlighted the variability of each tour for successive collections, in terms of traveled distance, number of visited clients and average service time. The waste fill level availability for each bin located in each site will help to prioritize clients that needs a waste collection from others where service could be postponed.

In the next section, we present data processing related to deployed sensors.

### 5.3. Sensors

**Figure 25:**
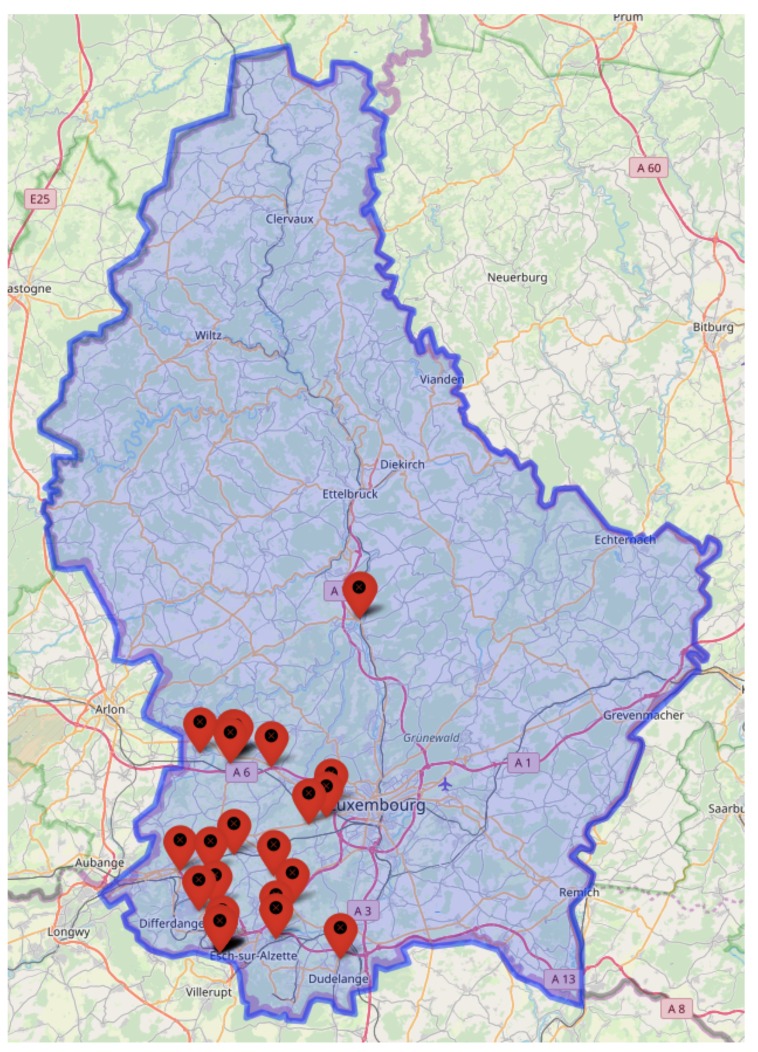
Physical deployment: 47 sensors were integrated in 35 distinct sites.

To measure the impact of SWAM on PoL’s daily operations, we deployed a set of sensors (see Section 4.3) on clients that belong to a specific waste collection tour (i.e., Monday for the south tour, see Figure 25).

**Figure 26:**
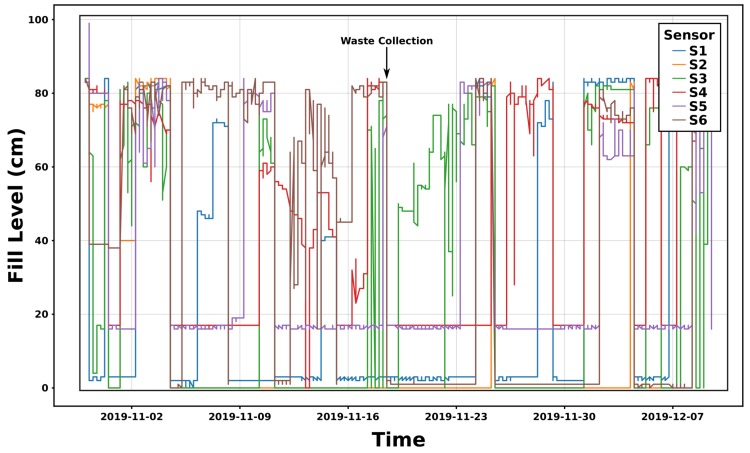
Raw data on a site composed by 6 bins.

One solution consists of a full deployment (one sensor per bin) but this would have definitively a significant high cost for PoL. In our use-case, a client may have a maximum of six bins dedicated to domestic waste. Waste production profiling from historical data will help to use less sensors (1 or 2 sensors per site with at least one sensor on the last bin based on the filling order, e.g., distance from the door of the technical room where they are stored). Each client was asked to respect this order. The sensor integration depends mainly on the bin maximum capacity (width W, length L and height H). The volume linked to the fill level is assumed to be a parallelepiped (rectangular cuboid). The waste volume is defined by V=W∗L∗F where F is the fill level (distance between the bottom of the bin and the average waste plan). Figure 26 provides an example of a fully equipped site with six bins (1100 L). In that configuration, the maximum F value reaches 88 cm.

Each sensor was configured with a time period of 30min between measurements only for the profiling phase (waste production for each activity). This period will be remotely updated after the completion of this phase in order to optimize the sensors’ lifetime. The selected sensor has a blind zone of 15cm. We can see in Figure 26 that each bin has its own waste production time evolution. A waste collection is highlighted with a black arrow. In fact, each full bin (i.e., where F almost reaches the maximum level) is emptied. Its filling level becomes insignificant (i.e., F = 0) for the next measurement. We note that some sensors never provide a zero value even after the waste collection (see sensors S4 and S5). An additional calibration has to be applied in order to normalize the raw values to the global range (0 to full level). Moreover, we observe many peaks that have already been filtered on Figure 26 due to multiple reflections (see Figure 10).

We presented the first results of the first phase of our sensor deployment in Luxembourg. We showed an example of one client site where six bins were equipped with ultrasonic sensors. The time series analysis of the measurements will help to profile the waste production and also to trigger a waste collection when defined thresholds were reached.

The next section provides discussions on our research studies.

## 6. Discussion

This paper is a preliminary study, which underlines a reality: in waste management but also in other sectors, the information collected by companies is mostly extremely limited. Cleaning and filtering efforts must be made in order to systematically remove anomalies. Going even further, a significant impact can only be achieved by employing connected technologies, offering a real time overview of the service to be provided. Other available metrics are, for the most part, uncorrelated. This opens up two discussions.

### 6.1. Data Aggregation–Correlation

The main difficulty of this research study was to correlate multiple data resources coming from non interoperable information systems. In fact, a waste collection on a single site consists of the emptying of one or multiple bins within PoL’s truck. We detected the arrival on site and the departure of the truck in respect with the GPS tracker installed on it and connected through the Winfleet API. The service time on site was proposed to be used in the SWAM optimization module. Its actual value depends on many factors, such as:

- The real number of processed bins. It can be retrieved with ultrasonic sensors prior to the waste collection or at the end of the day when interventions are updated into the ERP database.
- The site configuration (indoor/outdoor, doors, access ramp, etc.). Unfortunately the distance and the time needed to travel from the truck to the bin location (and vice versa) are not known.

The sensor integration has a significant impact on the fill level measurement. It should be the same for each bin capacity. The selected sensors provide multiple configuration angles for side/lid integration. However these angles can present minor differences from one sensor to another one, generating errors on measurements. Moreover, errors are amplified when multiple reflections occur. We assumed that the fill level is homogeneously distributed in a container. This is not true for large containers. A calibration will be designed in order to adapt the fill level computation according to each bin capacity and dimension, and also sensor integration. We observed that raw sensor data present discrete peaks that have to be filtered out (see Figure 26).

The monitoring frequency was fixed to 30min between successive measurements during the first deployment phase related to client’s profiling. It should be changed to 2 measurements per day with an optimal time selection in order to guaranty an acceptable lifetime (almost 5 years). In case of missing values (e.g., no measurement due to communication errors, or inconsistent data due to multiple reflections), interpolation will be used to make data coherent and also enable prediction at near future (24-48h) based on historical data.

Finally, many sites present several bins. We equipped them on a partial or total basis (for instance 6 bins on 6, or 2 bins on 6 with at least one sensor on the last bin). We will investigate the impact of the number of sensors on each site when data will be available. When the cleaned ERP data will be available, we will also analyze the correlation between weight data (truck), volume data (ultrasonic sensor) and service time data (Winfleet).

### 6.2. Optimization

As explained in Section 3.1, the goal of the SWAM project is the development of a system to optimize waste collection. To achieve this objective, we identified two main challenges: *(1)* selection of clients which should be included in a collection tour, *(2)* computation of the best suited tour to visit the selected clients. Traditionally the objective target of such systems is the minimization of the travel distance or a cost value, while respecting the constraints of the waste collection company and its clients [9]. To highlight the significance of client selection, it is important to know that the waste collection truck encounters one of three scenarios at each client site:

- **Too early:** the truck arrives too early and the bins are not filled to an appropriate level. This is not desirable for the waste collection provider because the company invested its resources in a service which could be performed at a later point in time.
- **Too late:** the truck arrives too late and the bins are filled to overflowing. This is not desirable for for both the client and the waste collection provider. The waste collection provider now needs to invest more service time to collect all the waste and the client is unsatisfied with the provided service quality.
- **Just in time:** the truck arrives and the bins are filled to an appropriate level. This is the desired scenario.

This suggests that the waste collection provider needs more detailed information about the filling level of the clients’ bins. As described in Section 4.3 we deployed ultrasonic sensors to measure the exact filling level of a subset of all the clients. The cost of these sensors are a considerable investment for a waste collection provider. With this in mind, we need to justify that the sensors are providing an additional value. To verify their value, we propose different incremental steps of optimization. A higher level of optimization is using more advanced techniques and thus should result in a better performance. We propose three steps of optimization:

- Optimization based on static information (see Section 5.1) from client contracts (e.g., allocation and clustering of clients by using their site’s location and their collection frequency).
  - *Expectation:* This first optimization should significantly reduce the traveling distance to complete waste collection tours. However this step should have no influence on reaching the **just in time** scenario.
- Optimization based on added historical data to form client profiles and predict their waste generation (see Section 5.2).
  - *Expectation:* With the use of historical data about the amount of waste collected at each client, it should be possible to identify weekly and/or seasonal patterns for waste generation. The accuracy of the resulting estimations needs to be examined, but overall this should lead to a significant improvement compared to the first step.
- Optimization based on added sensor measurement data (see Section 5.3).
  - *Expectation:* Through the use of the near real-time measurements of the bins’ filling level, the accuracy of waste generation prediction can be improved. This should lead to further performance improvements compared to the second step.

By using an incremental approach to develop and implement an optimization system, we can benchmark the performance of the individual steps. Furthermore, these measures can be used to compare the performance of the different optimization steps. By adding information about the implementation cost of the individual steps, we can compute which level provides the best *return on investment*.

Overall, the collected data sets and the generated features open up multiple possibilities to optimize the waste collection. As part of the SWAM project we are planning to follow the presented increment approach to facilitate the comparison between solutions of different complexity.

## 7. Conclusions and Future Work

In this paper, we presented SWAM, a waste management platform for business, and SWAM-DMS its data management system that relies on ultrasonic sensors deployed all around Luxembourg. SWAM-DMS collects PoL’s data flows and is accessible via an interoperable API. It integrates an analytics component to infer relationships between variables, filtering mechanisms to ensure the data’s consistency and reliability and prediction mechanisms to interpolate potential missing values or extrapolate future trends.

We extracted many features from these data resources (ERP, fleet management, and the sensor network) and analyzed them. We highlighted a few metrics from a PCA decomposition that enables profiling the waste production of each client based on their activity. This information will help to include a client into a waste collection tour, according to the SWAM optimization module. We presented our algorithms that extract waste collection tour from GPS tracking datasets and perform service time profiles for each client’s site. We showed that the current collection paths can be optimized by minimizing the total traveled distance and the service time on site according to each client’s profile and also maximizing the number of clients obtaining a service, and the collected weight. We finally deployed 47 sensors on 35 distinct sites.

In future work, the generated filling level data will be analyzed and correlated with collected weight and actual waste collection paths. We also intend to extend the analysis made in this paper by implementing predictive mechanisms on sensor data, which would require a substantial amount of historical data but has the possibility to understand the variability of seasonal activities. We also plan to further work on route optimization for customer satisfaction. Finally, it is planned to deploy more than 200 sensors all around Luxembourg by the end of 2020: this will lead to more in-depth studies.

## Figures and Tables

**Table 1:** Differences between Public and Commercial Waste Collection.

Property	Public Waste Collection	Commercial Waste Collection
Client scope	Private households	Companies, restaurants, schools
Client waste generation	Homogeneous	Heterogeneous (1000–10,000L per week)
Collection scope	Communities	Country-wide/regions
Distance between clients	Short (neighbors)	Significant, but can be diverse
Bin placement	On the pavement	Somewhere on the client site

**Table 2:** A comprehensive analysis based on the prior art on SWC (Dynamic Scheduling DS, Dynamic Routing DR).

Study	Bin Location	Waste Type	Sensors	GPS	Implementation	DS	DR
[13]	Outdoor	Glass; Plastic; Paper; General Waste	Weight & Capacity	No	Simulation	No	No
[14]	Outdoor	Organic, Plastic/ Paper/Bottle, Metal	Capacity	No	Simulation	Yes	Yes
[15]	Outdoor	General	-	Yes	Simulation	No	No
[16]	Outdoor	General	Capacity	No	Simulation	Yes	Yes
[17]	Outdoor	Plastic	Weight & Capacity	No	Simulation	No	No
[18]	Outdoor	General	Capacity	Yes	Real	No	No
[19]	Outdoor	General	Capacity	No	Simulation	Yes	Yes
[20]	Outdoor	General	Capacity	Yes	Simulation	Yes	Yes
[21]	Outdoor	General	Capacity	Yes	Simulation	No	Yes
[22]	Indoor	General	Capacity	No	Real	Yes	Yes
[23]	Outdoor	General	Weight	Yes	Real	No	No

**Table 3:** Correlation matrix: Activity vs Cluster (k-mean).

Activity	Cluster 1	Cluster 2	Cluster 3	Cluster 4
Retail Business	0.302	0.581	0.023	0.093
Construction	0.531	0.406	0.031	0.031
Housing Activity	0.400	0.300	0.200	0.100
Restaurant	0.185	0.481	0.074	0.259

**Table 4:** Correlation matrix: Activity vs Cluster (PAM).

Activity	Cluster 1	Cluster 2	Cluster 3	Cluster 4
Retail Business	0.279	0.511	0.093	0.116
Construction	0.531	0.219	0.219	0.031
Housing Activity	0.400	0.000	0.300	0.300
Restaurant	0.185	0.222	0.296	0.296

## Abbreviations

The following abbreviations are used in this manuscript:

DR	Dynamic Routing
DS	Dynamic Scheduling
ERP	Enterprise Resource Planning
GPS	Global Positioning System
IOT	Internet Of Things
MDPI	Multidisciplinary Digital Publishing Institute
NACE	Socio-professional category
PAM	Partitioning Around Medoids
PCA	Principal Component Analysis
PoL	Local waste company
SWC	Smart Waste Collection
SWAM	Smart WAste Collection SysteMs
SWAM-DMS	SWAM Data Management System
